# Cardiometabolic Health Intervention Using Music and Exercise (CHIME) Delivered via Telehealth to Wheelchair Users: Protocol for a Randomized Controlled Trial

**DOI:** 10.2196/57423

**Published:** 2025-01-15

**Authors:** Yumi Kim, James H Rimmer, Byron Lai, Robert Oster, Rachel Cowan, Hui-Ju Young, Gordon Fisher, Younguk Kim, John Giannone, Jereme D Wilroy

**Affiliations:** 1 Department of Physical Medicine and Rehabilitation University of Alabama at Birmingham Birmingham, AL United States; 2 Department of Occupational Therapy University of Alabama at Birmingham Birmingham, AL United States; 3 Division of Pediatric Rehabilitation Children's Hospital of Alabama Birmingham, AL United States; 4 Department of Preventive Medicine University of Alabama at Birmingham Birmingham, AL United States; 5 Department of Human Studies University of Alabama at Birmingham Birmingham, AL United States

**Keywords:** exercise, physical activity, wheelchair user, telehealth, disability

## Abstract

**Background:**

Wheelchair users live predominantly sedentary lifestyles and have a substantially higher risk for cardiometabolic disease and mortality compared to people without disabilities. Exercise training has been found to be effective in improving cardiometabolic health (CMH) outcomes among people without disabilities, but research on wheelchair users is limited and of poor quality.

**Objective:**

The primary aim of this study is to examine the immediate and sustained effects of a 24-week, telehealth, movement-to-music cardiovascular (M2M-C) exercise program on core indicators of CMH among adult wheelchair users compared to an active control group. The secondary aim is to explore the beneficial effects of M2M-C exercises on cardiovascular capacity, physical activity, and quality of life. Intervention components include tailored exercises and remote performance monitoring, delivered via live videoconference training by a telecoach and asynchronous videos.

**Methods:**

This study’s design is a parallel-arm randomized controlled trial enrolling 132 physically inactive adult wheelchair users with poor cardiometabolic profiles. The M2M-C intervention group involves 24 weeks of virtual live and monitored home exercise training (3×/wk, 15-40 min/session), followed by a 12-week maintenance period where participants have access to an online media library of exercise videos. The control group involves 36 weeks of self-guided exercise through access to a media library of exercise videos, including videos for range of motion, muscle strength, and balance. The primary outcomes are cardiometabolic indicators of health, and assessors are blinded.

**Results:**

Recruitment procedures started in January 2024 with the first participant enrolled on March 18, 2024. All data are anticipated to be collected by November 2027, and the main results of the trial are anticipated to be published by February 2028. Secondary analyses of data will be subsequently published. A total of 16 participants have been recruited as of paper submission.

**Conclusions:**

The knowledge obtained from this trial will provide evidence to inform exercise prescriptions aimed at improving CMH among adult wheelchair users.

**Trial Registration:**

ClinicalTrials.gov NCT05606432; https://clinicaltrials.gov/study/NCT05606432

**International Registered Report Identifier (IRRID):**

DERR1-10.2196/57423

## Introduction

### Background

People with physical disabilities have higher risks of cardiometabolic disease than the general population. For example, studies have reported that as many as one-third of people with spinal cord injury (SCI) may be diagnosed with cardiometabolic disease (ranging from n=41, 29% to n=222, 34.1%), which is nearly twice as prevalent as reported in people without SCI [1-3]. Another study found that adults living with cerebral palsy or spina bifida are 52% more likely to experience any type of cardiometabolic morbidity than adults without cerebral palsy or spina bifida (n=15,302) [4]. Meta-analyses have demonstrated that conventional aerobic exercise, such as running, walking, and cycling at a moderate-to-vigorous intensity, are effective in managing indicators of cardiometabolic health (CMH) in the general population [5-9]. However, people with physical disabilities, particularly wheelchair users, are typically unable to engage in conventional modalities of exercise long enough to obtain CMH benefits. Wheelchair users include users of any wheeled devices for their primary means of mobility, such as manual and powered wheelchairs or motorized scooters, and may include individuals in a variety of disability groups, including those with SCI, cerebral palsy, spina bifida, multiple sclerosis, or limb loss. A total of roughly 5.5 million wheelchair users in the United States would benefit from an accessible aerobic exercise program to reduce their cardiometabolic disease risk [10].

However, there is limited evidence that aerobic exercise improves CMH among wheelchair users. Most evidence is observational and nearly all randomized controlled trials (RCTs) have small sample sizes limited to a single population (eg, those with SCI). There are no large confirmatory RCTs that demonstrate that aerobic exercise improves CMH in adult wheelchair users. A recent meta-analysis of 3 RCTs, with samples of 15-21 adults with SCI, determined that 6-weeks of aerobic exercise improved CMH among adults with SCI (ie, decreased fasting glucose and insulin and increased fasting high-density lipoprotein cholesterol fraction) [11]. Another meta-analysis of 16 pre-post and controlled trials with sample sizes of 5-20 adults with SCI determined that vigorous exercise training improved cardiorespiratory fitness in adults with SCI [12] (ie, increased peak power output, muscular strength, and time to fatigue) but did not improve CMH risk factors, such as blood pressure (BP), cholesterol, and insulin sensitivity. Noted limitations of these studies included small sample sizes, inclusion of participants already within healthy ranges for CMH at baseline, lack of a control group, use of laboratory-based training, and inclusion of a single population (those with SCI). Therefore, the finding that CMH measures did not improve must be interpreted with caution.

Interventions that have sought to improve physical activity levels have also failed to improve CMH among wheelchair users. The largest physical activity trial among wheelchair users (n=128), Workout on Wheels, increased self-reported physical activity by 17 min/wk, with no significant between-group differences in cardiorespiratory fitness or CMH measures [13]. Other recent lifestyle interventions (eg, behavioral coaching) have similarly failed to elicit significant CMH benefits among people with disabilities, even while achieving large sample sizes [14]. Thus, the exercise doses or intensity studied may be suboptimal for improving CMH among people with physical disabilities. Despite failures to improve CMH, many of these physical activity trials have informed the creation of the most recent guideline related to CMH for people with SCI [15,16]. Collectively, there is an urgent need for well-designed interventions that can provide an effective exercise dose for people with disabilities, including wheelchair users, while obtaining evidence for improving CMH through physical activity.

### Rationale for Telehealth Exercise Program

A telehealth exercise program was developed to overcome the barriers of participation in exercise requiring individuals to leave their homes and use expensive equipment. This study uses an exercise program that includes various movement patterns that are coupled with elements of music and imagery, referred to as movement to music (M2M) [17]. The M2M program uses a range of different muscle groups allowing for a safer form of exercise than arm ergometry. Moreover, M2M incorporates a theory-driven telehealth delivery protocol that removes the most common barriers to participation in community and clinical exercise training studies (ie, cost, transportation, and time) [18,19]. The design of the movement-to-music cardiovascular (M2M-C) intervention is based on four key elements of social cognitive theory: self-efficacy, self-regulation, outcome expectations, and social support. These components are essential for promoting positive health behavior change. To effectively integrate these principles, the M2M-C program provides a structured, individualized, and progressively challenging exercise routine. Participants receive regular feedback and personalized guidance from M2M-C instructors and telecoaches, which helps build confidence and maintain motivation. In addition, the incorporation of music enhances movement quality and motor coordination [20] while serving as a powerful motivational tool [21]. By increasing enjoyment and improving mood, music makes exercise more appealing and engaging. This approach is consistent with the broader goal of promoting sustained physical activity among wheelchair users by addressing both psychological and physical barriers to exercise adherence.

To test whether the M2M program can improve CMH outcomes among wheelchair users, this study uses a parallel-group RCT with blinded assessors. The intervention group will use the M2M program, composed primarily of cardiovascular exercises (M2M-C), which will be compared to an active control condition using prerecorded non-M2M exercise videos. The M2M-C exercises were informed by disability exercise guidelines and reviews of relevant studies [22]. This study is referred to as the Cardiometabolic Health Intervention Using Music and Exercise (CHIME) study. The primary aim is to examine the effects of a 24-week, synchronous M2M-C program on core indicators of CMH in adult wheelchair users with ≥2 cardiometabolic risk factors. The secondary aim will explore the beneficial effects of M2M-C exercises on cardiovascular capacity, physical activity, and quality of life. We hypothesize the M2M-C intervention will yield both immediate and sustained CMH outcomes among adult wheelchair users with poor cardiometabolic profiles.

## Methods

### Study Design

This study’s protocol follows the SPIRIT (Standard Protocol Items: Recommendations for Interventional Trials) guidelines [23,24].

### Aims and Design of this Study

This study’s design includes three aims. The primary aim examines the immediate effect of a 24-week, synchronous M2M-C training protocol compared to a 24-week, asynchronous activity control on core indicators of CMH among 132 adult wheelchair users with ≥2 cardiometabolic risk factors. The secondary aim examines the immediate effect of M2M-C training compared to the active control condition on cardiovascular capacity, physical activity, and quality of life. The first tertiary aim evaluates the sustained effects of M2M-C training (24 to 36 wk) on physical activity participation. A second tertiary aim evaluates the potential response heterogeneity in CMH, cardiovascular capacity, and physical activity outcomes using prespecified moderator variables (eg, age, sex, body composition, diet, or ethnicity) to understand for whom the intervention is most effective.

This study’s aims will be tested using a parallel-group, assessor-blinded RCT design. For primary and secondary aims, we hypothesize that a telehealth M2M-C program will lead to significantly greater improvements in CMH outcomes, peak oxygen consumption (VO_2peak_), physical activity level, and quality of life from baseline to 24 weeks compared to controls. For the tertiary aim 1, we hypothesize that M2M-C participants will maintain physical activity levels from week 24 to week 36.

### Participants

#### Recruitment

We are recruiting participants who receive health care from a large medical center residing in the southern United States through direct referrals from health care professionals (eg, physicians or therapists) and distribution of study materials (eg, flyers, newsletters, or advertisements) to various clinics, hospitals, and medical rehabilitation service providers.

#### Eligibility Criteria

Adult wheelchair users who do not participate in health-enhancing volumes of exercise [16] and have ≥2 cardiometabolic risk factors are recruited for this study. Potential participants are prescreened for the eligibility criteria via telephone screening, medication list review, and physical screening. The onsite, physical screening to confirm eligibility occurs in the baseline visit.

Textbox 1 lists the specific participant eligibility criteria. Participants undergo a 2-stage screening process. The first stage, eligibility screening, is undertaken during a scripted phone screening by the project coordinator. The presence of CMH risk factors [25] is initially screened based on the self-reported height and weight to estimate the BMI and waist circumference, as well as the physician-prescribed medications that are relevant to CMH risk factors (eg, are you taking any medications related to increased BP? Has your doctor ever expressed concerns about your cholesterol level?).

**Inclusion and exclusion criteria.**

**Inclusion criteria**

- Full-time or part-time use of a wheelchair device (manual wheelchair, power wheelchair, or electric scooter) self-reported as the primary means of mobility
- Aged ≥18 years
- Able to use arms to exercise
- Obtaining <90 minutes of moderate-to-vigorous intensity exercise per week in the last month
- ≥2 cardiometabolic risk factors:
  Elevated waist circumference: ≥102 cm in men and ≥88 cm in women
  Elevated triglycerides: ≥150 mg/dL
  Reduced high-density lipoprotein cholesterol fraction: <40 mg/dL in men, <50 mg/dL in women
  Elevated blood pressure (BP): ≥130 mm Hg systolic BP or ≥85 mm Hg diastolic BP
  Elevated fasting glucose: ≥100 mg/dL
- No contraindication to participate in moderate-to-vigorous intensity exercise as informed by physician’s medical clearance for exercise
- Not currently participating in a structured exercise program
- Able to converse and read in English

**Exclusion criteria**

- Medically unstable to perform the prescribed home exercise as determined by their physician
- High-level tetraplegia and unable to use arms to exercise
- No internet access was determined via self-report and internet speed test
- Positive pregnancy test
- Other physical conditions that can potentially prevent them from participating in an exercise routine (eg, pressure sore and open wound)

**Justification for exclusion criteria**

- Exclusion of pregnant individuals: The use of dual-energy x-ray absorptiometry for body composition analysis involves minimal radiation exposure. However, to eliminate any potential risk to the fetus, pregnant individuals will be excluded.
- Exclusion of individuals with medical instability: To minimize the risk of adverse events during the intervention, participants must be medically stable and have clearance from a health care provider to engage in moderate-to-vigorous intensity exercise.
- Internet access requirement: As the intervention is delivered via a tele-exercise platform, participants must have reliable internet access to ensure they can fully engage in the program, participate in live sessions, and receive remote monitoring and feedback.

The second screening stage involves a review of participation medications (self-reported) and a physical examination by laboratory staff. The medication list is obtained before the baseline visit using a secure electronic survey, and this study’s staff reviews and verbally confirms them during the baseline visit. If the potential participants currently take ≥2 medications related to CMH risk factors, then they can proceed to the baseline physical examinations. Otherwise, they proceed to physical screening in the following order: manual BP, tape-measured waist circumference, and fingerstick blood sampling for triglycerides, high-density lipoprotein cholesterol fraction, and glucose using the lipid analyzer (Cholestech LDX, Abbott). If the potential participants meet the eligibility criteria with BP and waist circumference, fingerstick blood sampling is omitted.

### Ethical Considerations

Ethical approval for this study was obtained from the University of Alabama at Birmingham Institutional Review Board on December 22, 2022 (IRB-300009718). This study’s protocol adheres to all relevant ethical guidelines for research involving human participants, including medical records and patient information.

Informed consent will be obtained from all participants before the baseline visit or during the baseline visit. During the consent process, participants will receive detailed instructions about this study, including its objectives, procedures, potential benefits, and any associated risks or discomforts. They are also informed of their rights, including the ability to withdraw from this study at any time, to ensure that their participation is completely voluntary. This comprehensive approach is designed to ensure that all participants fully understand this study before providing their consent.

To protect participants’ privacy, all data collected will be anonymized by assigning each participant with a study number to ensure that no personally identifiable information is directly linked to the data. Demographic, personal, and exercise test data will be securely stored in REDCap (Research Electronic Data Capture; Vanderbilt University) and Box (Box, Inc), which are secure, password-protected databases. Access to these data will be limited to authorized research personnel, and all information obtained during this study will be used for research purposes only.

Participants will receive up to US $830 for their participation in this study. Compensation will be provided in stages, with participants receiving US $150 at the baseline assessment, US $200 at the 12-week assessment, US $250 at the 24-week assessment, and US $150 at the 36-week assessment. In addition, participants will receive US $40 for each of the two interview sessions to ensure fairness and transparency in recognition of their time and effort.

### Power Analysis and Sample Size

We plan to recruit 132 participants into this study (66 per treatment group), with the expectation that at least 100 (50 per group) will complete this study. We anticipate an attrition rate of 23% based on the average attrition rate between the largest home-based behavioral interventions among wheelchair users (33%) [13] and our scoping review of exercise trials for people with various disabilities (13%) [14]. Due to the nature of the proposed exercise training involving one-on-one training and supervision, we believe that the role of exercise training coaches in this study will lead to lower attrition rates as it has in our past studies [26].

Power calculations were performed using nQuery (version 8.5; Statsols). For the primary outcome of CMH, we obtained SDs of biomarkers of this outcome from our pilot study among individuals with SCI (6-wk arm crank exercise training), including SDs of 43 mg/dL for total cholesterol, 4.3% for body fat, and 22.4 mg/dL for triglycerides [27]. Assuming a final sample size of 50 participants per group, a 2-sided 2-group *t* test (2-tailed), and a significance level of .05, we will have at least 80% power to detect differences of 24.4 mg/dL for total cholesterol, 2.5% for body fat, and 12.7 mg/dL for triglycerides (and greater) between the intervention and control groups as being statistically significant. This is equivalent to detecting an effect size (Cohen *d*) of 0.566 (a moderate effect size) as statistically significant between the two groups.

### Outcomes

**Figure 1:**
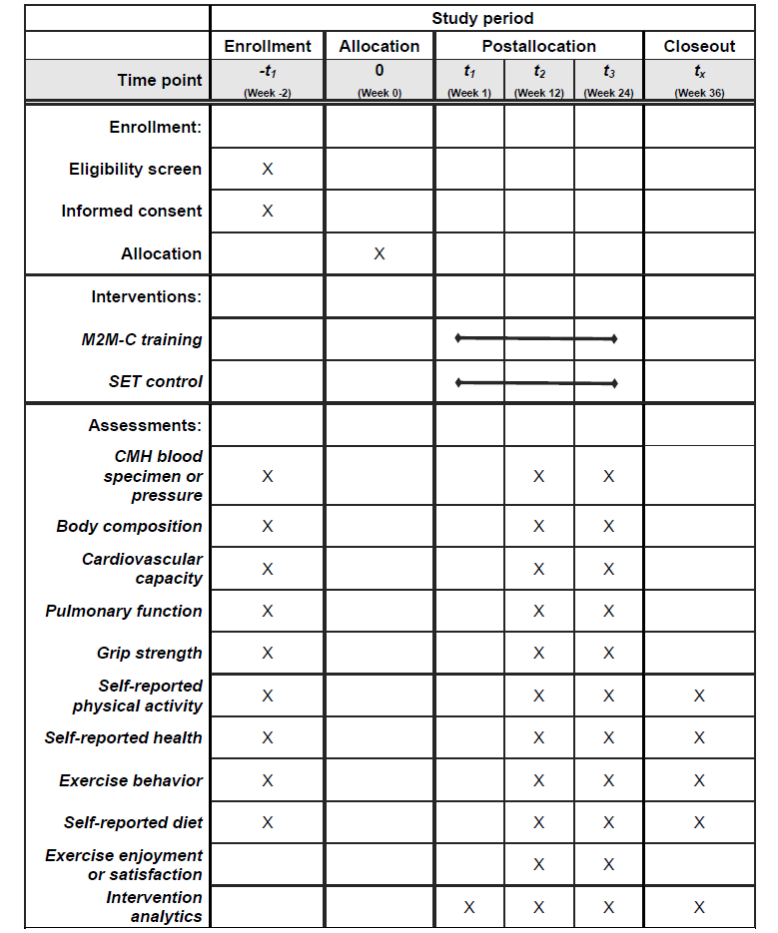
Example template of recommended content for the schedule of enrollment, interventions, and assessments. CMH: cardiometabolic health; M2M-C: movement-to-music cardiovascular; SET: standard exercise training.

Outcomes are assessed at baseline, midintervention (12 wk), postintervention (24 wk), and follow-up (36 wk) time points. Physiological outcomes are assessed by anonymous-to-treatment assessors. The assessors are not involved in random assignment or the intervention delivery and do not directly communicate with the M2M instructors and assistant health coaches about participants. Data collection with physiological outcomes is conducted at a local research facility affiliated with a major medical center. Self-reported outcomes are administrated via a secure electronic database, referred to as REDCap. Online platform analytics are used to assess heart rate (HR) and video minutes viewed and stored in the cloud. Figure 1 shows the schedule of assessments.

### Primary Outcome Measures

CMH outcomes are assessed via venipuncture blood specimen, brachial cuff BP, and body composition.

- High sensitivity C-reactive protein (hsCRP; mg/L): hsCRP is a critical marker of inflammation that contributes to proinflammatory and prothrombotic elements of cardiovascular risk. A single hsCRP measure is a strong predictor of myocardial infarction or coronary heart disease mortality and several other diseases of the circulatory system in people without a history of such conditions [28]. Changes in hsCRP may occur from as early as 8 weeks of exercise [29].
- Hemoglobin A1c (HbA1c, mmol/mol): HbA1c is a measure of red blood cell mean hemoglobin glycation over the previous 3 months. Exercise interventions without a dietary component should yield a small to moderate effect on HbA1c after a month of training [30].
- Fasting insulin (μIU/mL): A high fasting insulin level indicates the presence of insulin resistance and whether an individual shows glucose intolerance. Exercise interventions without a dietary component should yield a small beneficial change in fasting insulin levels after a month of training [30].
- Fasting glucose (mmol/L) and homeostatic model assessment of insulin resistance: This is calculated as [fasting insulin (μU/mL) × fasting glucose (mmol/L)]/22.5 [31].
- Fasting triglycerides (mg/dL): A triglyceride level >150 mg/dL, is largely supported as an indicator of cardiovascular risk [32,33]. Exercise interventions without a dietary component should yield a small beneficial change in triglyceride levels after a month of training [30], even among people with normal triglyceride levels [34].
- Fasting cholesterol (mg/dL): Abnormalities in the lipid profile, including elevated total and LDL cholesterol as well as decreased HDL cholesterol, are predictors of future cardiovascular disease among young and middle-aged people [35,36]. Exercise interventions without a dietary component should yield a small effect after 1 month [30].
- BP (mmHg): Moderate-intensity exercise has been shown to reduce BP [37].
- Waist circumference (cm): The waist circumference is measured at the level of the superior border of the iliac crest, above the umbilical level, and at the midline level [38] using a Gulick tape.
- BMI (kg/m^2^): Dual-energy x-ray absorptiometry (DXA) scan is used to measure weight-adjusted BMI, in addition to total mass, lean mass, and fat mass. It is evident that people with SCI often have elevated waist circumference (≥94 cm), but their BMI is less than 30 kg/m
  2
  [39].

### Secondary Outcome Measures

Secondary outcomes include cardiovascular capacity, pulmonary function, grip strength, and self-reported physical and psychosocial health outcomes.

- VO_2peak_ (ml/kg/min): VO_2peak_ is measured during a graded exercise test as an indicator of cardiovascular capacity using an arm ergometer (Lode) and open circuit spirometry with a metabolic cart (TruOne, ParvoMedics). Arm ergometers are considered the gold standard modality for exercise testing among people with disabilities who use wheelchairs or cannot run or cycle for prolonged periods [40,41]. Before starting the test, participants rest for 3-5 minutes. They are then instructed to maintain a pace between 55 to 65 revolutions per minute throughout the testing period. The participant starts their test with 2 minutes of warm-up without any resistance, and then the resistance is increased every minute by 10 watts until the participant reaches volitional fatigue or subjective discomfort (eg, shortness of breath and chest pain) or achieves 2 or more of the following termination criteria: age-predicted maximum HR of more than 85%; rate of perceived exertion (RPE) of 8 or more on a 0-10 scale; respiratory energy exchange ratio of 1.1 or higher; or plateau in oxygen consumption [42]. HR and oxygen consumption is measured continuously.
- FEV1 (forced expiratory volume over 1 second) and FVC (forced vital capacity; liters): Pulmonary function is measured by (1) how much air the participant exhales quickly, and (2) how much air can be expelled after taking a full breath via a portable spirometer (MIR Spirobank). These will be measured 3 times, with a 1-minute resting period between each trial during the testing session.
- Grip strength (kg): Grip strength is measured using a hand-held dynamometer (Jamar Hydraulic Hand Dynamometer, Lafayette Instruments) for both hands, with 3 trials conducted for each hand and a 1-minute resting period between each trial.
- Self-reported physical activity (min/wk): The volume of physical activity is measured using the Leisure-Time Physical Activity Questionnaire for SCI [43].
- Self-reported physical and psychosocial health: Various aspects of physical and psychosocial health are measured using standardized questionnaires by the National Institutes of Health PROMIS (Patient-Reported Outcomes Measurement Information Systems) [44]. This includes Pain Intensity, Pain Interference, Fatigue, Sleep Disturbance, Global Health, Ability to Participate in Social Roles and Activities, Depression, Anxiety, and Physical Function.

### Moderating Outcome Measures

The moderating outcome measures are as follows:

- Demographics and clinical characteristics of participants (eg, age, sex, ethnicity, diagnosis, or medications)
- Exercise behavior measured by Exercise Self-efficacy Scale [45,46], and Exercise Goal Setting and Planning Scale [47] based on Social Cognitive Theory constructs
- Self-reported diet using the Rapid Eating Assessment for Participants Shortened Version [48,49]
- Exercise enjoyment using the Physical Activity Enjoyment Scale [50] and satisfaction via qualitative, semistructured one-on-one interview
- Exercise session duration based on attendance rate and video minutes viewed via online platform analytics
- Exercise intensity based on minutes exercised at moderate-to-vigorous intensity using an optical HR monitor (Polar Verity Sense, Polar) and RPE scale 0-10: The RPE is periodically asked during the exercise session to adjust the intensity and at the end of the exercise session to obtain the overall exertion level. The talk test is used as a secondary measure, primarily to ensure that participants stay in the moderate-to-vigorous intensity training zone and that RPE is related to aerobic strain as opposed to anaerobic. Additionally, this is an important indicator of exercise intensity, especially for individuals with a blunted exercise HR response.

### Random Assignment

After participants are determined eligible and all baseline assessments are complete, they are randomized into 1 of the 2 groups, M2M-C or control (n=66 per group), with a 1:1 allocation ratio. The randomization sequence is generated and only known by the project statistician using a computer-generated random schedule in permuted block (SAS; version 9.4; SAS Institute Inc). The block size is unknown to intervention staff.

### Intervention Arm—Home-Based, Synchronous Aerobic Exercise Training Using M2M

The prescriptive exercise guideline in this proposed study was generated through a critical review of the relevant literature. To determine an appropriate exercise dose, we used data from 16 studies of moderate-intensity aerobic exercise included in a recent meta-analysis on CMH effects of exercise training in the general population [22]. Specifically, we calculated the total prescription (average prescription per week × intervention duration) for each study and the overall average. We determined that an aerobic exercise training dose of approximately 2400 minutes (40 h) over 6 months would be necessary for CMH benefits. We then spread the minutes across a 24-week intervention period for this trial with wheelchair users to ensure adequate time to safely and progressively reach the prescribed dose. The session structure and exercise minutes for each week are presented in Table 1, and the intervention components are summarized in Table 2.

**Table 1:** Movement-to-music cardiovascular session structure and exercise in minutes for each week.

Week	1	2	3	4	5	6	7	8	9	10	11-24
Checking in	5	5	3	3	3	3	3	3	2	2	2
Range of motion	5	5	5	5	5	5	5	5	5	5	5
Aerobic	15	15	20	20	25	25	30	30	35	35	40
Cool down	5	5	5	5	5	5	5	5	5	5	5
End discussion	5	5	5	5	5	5	5	5	3	3	3
Exercise min/session	25	25	30	30	35	35	40	40	45	45	50
Total min/session	40	40	45	45	50	50	55	55	60	60	60

**Table 2:** Summary of movement-to-music cardiovascular program features.

Feature			Description
**Prescription**			2400 minutes of exercise across 24 weeks Progressive Individualized
**Frequency**			3 times per week
**Session duration**			25 to 50 minutes
**Intensity**			Moderate-to-vigorous Maximum heart rate at or above 70% Rate of perceived exertion between 3 and 7
**Intervention length**			24 weeks
**Setting**			Home
**Exercise mode**			Aerobic using M2M^a^
**Supervision**			
	Who	M2M instructors Assistant telecoaches	
	Mode	Remote telecoaching	
	Oversight	Zoom (Zoom Communications, Qumu Corporation) and TeleRehab app (JNP Enterprises LLC) Live, instruction sessions with M2M instructors Monitoring sessions with assistant health coaches using recorded live session videos	
**Meeting with the instructor or coach**			
	Weeks 1-8	1× M2M instructors 2× assistant telecoaches	
	Weeks 9-16	1× M2M instructors 1× assistant telecoaches 1× self-guided with no supervision	
	Weeks 17-24	1× assistant telecoaches 2× self-guided with no supervision	
**Equipment provided**			Computer tablets Tablet stand Polar heart rate monitor
**Intervention safety**			Zoom oversight Adverse event or serious adverse event reporting

^a^M2M: movement to music.

During the 24-week training phase, the M2M-C group receives 1:1 synchronous training from M2M-trained exercise instructors and assistant health coaches within a remotely coached, home-based setting using telehealth monitoring platforms (ie, Zoom, TeleRehab app). The intervention is administered 3 times per week, starting at 25 minutes per session and increasing by 5 minutes every 2 weeks to a maximum of 50 minutes. The exercise program focuses on improving cardiorespiratory fitness using a series of movement patterns accompanied by music. The program is tailored to an individual’s functional abilities (eg, use lower limb if able, slow vs fast tempo, level of trunk control) and preference of musical themes, including classical, country, decades (oldies), international, jazz or blues, and pop.

For the first 8 weeks of the training phase, participants attend 1 instructional session with an M2M instructor per week, and the live session is video-recorded via Zoom. Participants attend 2 monitoring sessions in the same week to repeat the exercise using the prerecorded video of the week with assistant telecoaches. For the second 8 weeks (wk 9-16), participants continue to attend 1 instructional session with an M2M instructor per week, and the live sessions are video recorded. Participants attend 1 monitoring session with assistant telecoaches and 1 self-guided session without any supervision. For the third 8 weeks (wk 17-24), participants no longer meet with M2M instructors. Using the recorded videos between weeks 11 and 16 (ie, 40 minutes per video; archived with no instructions), participants attend 1 monitoring session per week with assistant telecoaches and 2 self-guided sessions in the same week.

### Telehealth Monitoring Platform

The exercise intensity is prescribed at a moderate-to-vigorous intensity level, which is monitored through a web-based platform with physiologic devices to support remote telehealth supervision of the intervention. The platform, referred to as TeleRehab, includes an Android (Google LLC) app that is installed on a computer tablet with Bluetooth capability (Samsung FE S7, Samsung USA), which sends data and allows 2-way communications to a secure web server. HR data are recorded and transmitted for real-time viewing by both the user and research staff using an optic HR sensor (Polar Verity Sense, Polar). The device instructions from the manufacturer state that the sensor can be worn on the head, forearm, or upper arm. For the M2M-C exercise intervention, the HR sensor is placed on a thin, elastic sports headband and worn on the temple of the head. Based on internal testing among research staff (nonpublished findings), the head placement resulted in more accurate readings during intervention exercise than when the device was worn on the forearm or upper arm. The head placement avoided signal disruption caused by the rapid and consistent arm movements required for the exercise intervention. At the end of each session, the platform provides the summary of exercise minutes (ie, light or moderate-to-vigorous) based on the predetermined HR zone. For participants with blunted HR, RPE is used. The TeleRehab app was an upgraded version of one used previously in a tele-monitored feasibility exercise study among people with SCI [51].

### Control Arm—Home-Based, Asynchronous Standard Exercise Training

For ethical and engagement purposes, the control group receives access to a YouTube playlist of standard exercise training (SET) videos adapted for people with physical disabilities. They are advised a similar prescription as the intervention group of 3 exercise sessions per week. The SET program is based on the National Center on Health, Physical Activity, and Disability 14-Weeks to a Healthier You program launched in 2008. It involves various exercises that are performed in both standing and seated positions, including the upper and lower extremity range of motion (2 videos, 5 min each), muscle strength (3 videos, 5 min each), aerobic fitness (1 video, 5 min; 2 videos, 10 min each), balance (1 video, 2 min; 2 videos, 5 min each), and a cool down (1 video, 5 min) [52].

### Procedure

This study’s procedure is administered by a project coordinator with oversight by the principal investigator (PI) and coinvestigators and monitored through a fidelity monitoring plan. Our intervention fidelity plan is based on the best practices and recommendations from the Behavior Change Consortium [26]. These recommendations are intended to link theory and application across 5 primary study phases, including study design, provider training, monitoring and improving the delivery of the intervention, and monitoring and improving the enactment of intervention skills.

The project coordinator, who is specific to participant engagement, contacts interested participants via telephone, describes this study and its requirements, and conducts the screening based on this study’s inclusion and exclusion criteria. For eligible individuals, the project coordinator then obtains physician approval. Upon approval by the physician, the project coordinator then distributes the informed consent document electronically as a final step in enrollment. After completing the informed consent, a baseline self-report questionnaire packet and a current medication list form are sent electronically. The project coordinator follows up with a telephone call to schedule a baseline data collection visit and provide verbal and written instructions regarding the baseline testing procedure, completion of the questionnaire packet and medication list, and directions with parking information. The project coordinator contacts the participant by telephone 24 hours before the appointment as a reminder. Upon arrival at the adaptive human performance laboratory, the project coordinator reviews this study’s procedures with the participant and then initiates the baseline data collection. The baseline data collection is undertaken by treatment-anonymous research staff who will administer the second level of eligibility screening based on CMH indicators. The eligible participant then undertakes a series of physiological tests, and the composition of those measures takes approximately 2 hours.

Once the baseline assessment is completed, participants are randomly assigned to either the intervention or control conditions using a random numbers sequence with concealed allocation. The project coordinator unfolds the allocation information via this study’s database, communicates the condition of assignment with the participant and, if allocated to the M2M-C intervention, prepares the necessary equipment for program participation. The participant will also meet with instructors and assistant telecoaches. If allocated to the control condition (SET), the project coordinator prepares the tablet with access to the SET videos. Lastly, the project coordinator will schedule a goal-setting session to be completed within 2 weeks between the participant and PI. This is carried out for both groups.

Participants in both intervention and control groups receive a 15-minute, individual goal-setting session at each time point from the PI, who is trained in physical activity counseling. They discuss participant’s outcome expectations from the program and review the goal of the program based on the given exercise prescription. The participants are prompted to share any foreseen barriers to complying with the program and guided to develop a relapse prevention plan (eg, identifying activities and social support to reactivate participation; outlining consequences for continuing and discontinuing the program). Additionally, the participants are prompted to share additional health goals and receive guidance on making them specific, measurable, attainable, relevant, and time-bound.

The intervention and control participants complete the same measurement procedures at the 12-week (midintervention) and the 24-week (postintervention) time points. There is no physiological data collection at the 36-week follow-up, as the aim to be addressed is sustaining physical activity behavior.

Participants receive up to US $830 for completing the measures per assessment period, including baseline as well as 12-, 24-, and 36-week assessments, and 12- and 24-week interviews. Semistructured exit interviews are conducted at the 24-week assessment for the first 30 participants to identify opportunities for intervention improvement and refinement.

### Data Analyses

All data will be exported into and analyzed using SAS software (version 9.4 or later). Statistical tests will be 2-sided and will be performed using a significance level of 5%. Data analyses will follow intent-to-treat principles. Data will be initially examined for variations, outliers, errors, and patterns of missing values. Missing data will be inputted using multiple imputation techniques where necessary based on the assumption that the missingness mechanism is at random. Descriptive statistics, such as means, SDs, frequencies, and proportions, will be obtained for this study’s variables.

### Primary and Secondary Aims

We will analyze between-group differences (differences between the intervention and control groups) and within-group changes from preintervention to the 12-week midintervention (immediate effects of the intervention) time point. “Clinically significant improvement” was defined for each risk factor based on the minimum change deemed clinically important; for our purposes, “clinically significant improvement” equates to the following: approximately 10% decrease in total cholesterol, 30% reduction in triglycerides, and 5% reduction in body fat from the beginning of care to the most recent measure.

### Tertiary Aims

Our analyses will focus on only the within-group changes from the preintervention to the 12-week midintervention to the 24-week postintervention (sustained effects of the intervention) time point. For all 3 aims, comparisons of cardiometabolic indicators, measures of fitness, and scores of self-reported questionnaires between and within the 2 groups will be performed using generalized linear mixed model techniques, including mixed models repeated measures analyses. The specific covariance matrix, such as the unstructured covariance matrix, will be selected based on the final data. The Tukey-Kramer multiple comparisons test will be used as the post hoc test for pairwise comparisons of means. These models will allow us to assess the between-group effect, the within-group effect, and the group-by-time interaction. Covariates of scientific interest such as wheelchair use status (full-time vs part-time), medication use (use vs nonuse), and participant characteristics (eg, age, sex, race, and severity of disease) may be included in some of these models. Pearson (or Spearman, if needed) correlation analyses will be performed between pairs of study variables. Analyses of categorical variables will be performed using the Pearson chi-square test (or Fisher exact test, if needed). Baseline characteristics will be evaluated using the 2-group *t* test for continuous variables or the chi-square test for categorical variables. For all aims, missing data that is not rectified through ongoing review of source documents may be managed with multiple imputation, and the influence of the missing data assessed with sensitivity analysis. Continuous variables will be examined for normality of distribution using graphical techniques and tests of normality. Transformations will be performed for continuous variables that are not normally distributed, or nonparametric methods will be used for these variables.

Adherence and compliance data are logged onto the online training platform. Adherence and compliance rates will be assessed as proportions and their corresponding exact 95% CIs will be obtained. These rates will be obtained overall and separately by age, sex, body composition, and ethnicity. These rates will be compared separately for age group, sex, body composition, and ethnicity using the chi-square test or Fisher exact test if the assumptions for the chi-square test are not met.

We will consider the suggested outcomes as potential moderators and test effect modification by including appropriate interaction terms in our repeated measure of mixed models, including the severity of motor impairment (ie, PROMIS Physical Function 12a), clinical diagnosis (eg, SCI vs multiple sclerosis vs stroke), psychosocial metrics specific to readiness to change (eg, self-efficacy and goal setting skills), and other factors, such as wheelchair use status (full-time vs part-time), medication (use vs nonuse), and diet quality (healthy diet vs less healthy diet).

## Results

Recruitment procedures started in January 2024 with the first participant enrolled on March 18, 2024. All data are anticipated to be collected by November 2027, and the main results of the trial are anticipated to be published by February 2028. Secondary analyses of data will be subsequently published. A total of 16 participants have been recruited as of August 20, 2024.

## Discussion

### Principal Findings

The CHIME study proposes a parallel-group RCT for examining the 24-week effects of an evidence-based M2M program compared with a control condition for yielding immediate and sustained CMH outcomes among adult wheelchair users with poor cardiometabolic profiles. This study anticipates that after 24 weeks, participants in the M2M-C intervention group will show significant improvements in key cardiometabolic risk factors, such as reduced HbA_1c_ levels; lower fasting glucose and triglycerides; and decreased BP, waist circumference, and body fat percentage, compared to the control group. Furthermore, the M2M-C intervention is expected to improve health-related physical fitness, including measurements such as VO_2peak_, FEV1, FVC, and grip strength, in line with self-reported psychosocial health outcomes. In addition to the improvements in CMH and physical fitness, we anticipate that these benefits will be sustained during the maintenance phase (up to 36 wk). This suggests that wheelchair users may be able to adhere to telehealth-based exercise programs over the long term. If successful, this study will be the largest and sufficiently powered confirmatory RCT trial targeting CMH outcomes in this population. The prescribed exercise dose was informed by the review of the relevant literature [22]. If successful, this study will establish an evidence-based recommendation of adequate exercise dosing for adult wheelchair users with poor CMH profiles.

### Strength and Limitations

This study uses an evidence-based M2M program with modifications to focus on aerobic training and telehealth delivery [53,54]. The M2M program has been specifically designed and tested for safety and effectiveness for balance, walking endurance, and volume of physical activity among people with various disabilities (eg, multiple sclerosis, stroke, and SCI) [17,55,56]. Studies involving fitness training and traditional modes of exercise, such as riding a stationary bike and lifting weights, often result in low adherence rates for many people due to a lack of enjoyment or social interaction [57,58]. Several studies have reported that adding music improves exercise adherence [59] and enjoyment [60,61]. The enjoyment was a highlighted component of the M2M program in addition to its safety and effectiveness, and it has the potential to improve aerobic fitness and CMH when the exercise intensity is regulated by trained instructors.

This study has been designed to deliver quality exercise interventions in the convenience of the participant’s home with minimal exercise equipment. However, many exercise interventions often fail to be made available after research trials due to a lack of resources and support. To support the sustained exercise behaviors and gains, postintervention resources will be provided to study participants for their maintenance period (wk 25-36) and can be used after that time. The M2M-C intervention participants will have access to an online library of exercise videos, including their 24-week M2M-C videos, and the control participants will have access to the SET exercise videos. In addition, all participants will be introduced to the National Center on Health, Physical Activity and Disability (to receive continuous support through various health-related programs that are specifically designed for people with disabilities. Participants will be given the center’s website and email addresses, in addition to their toll-free hotline.

### Limitations

One of the major limitations of this study is that it relies on self-reported data for certain outcomes, such as physical activity, exercise behavior, and diet. Self-reported data can sometimes overestimate or underestimate actual behavior and can be affected by recall bias, where participants may not accurately recall their physical activity or exercise habits [62]. In addition, the time frame for these questionnaires is limited to a 1-week period, which may not fully capture participants’ typical physical activity and exercise patterns over time. To address these limitations, future studies could incorporate objective measures, such as wearable devices and activity trackers, to supplement self-reported data. These tools would provide a more comprehensive and accurate understanding of physical activity patterns and engagement, helping to mitigate the biases associated with self-reported data. An additional limitation of the trial is that participants are not blinded to treatment due to the nature of the intervention being an online exercise program.

We may experience problems with the participants adhering to this study’s protocol due to the requirement of onsite physiological data collection. Transportation is a well-documented barrier for people with disabilities, especially our target population who may require assistance with transferring in and out of a vehicle or obtaining accessible public transportation. To overcome this barrier, we have arranged a ride service that contains accessible vehicles. In addition, there may be some attrition during the maintenance period (wk 25-36) wherein there is no planned coaching and contact. Potential attrition is expected from this study but we have developed a plan to minimize it using the taper-down approach of coaching and contact during the 24-week training period.

Lastly, the current design of the trial involves intensive supervision of training by the M2M instructors and assistant health coaches, which is approximately 43 hours per participant over the 24-week training period (ie, 19, 16, and 8 hours for each 8-wk phase). The high volume of staff time to implement this study may hinder the scalability of the program in its current form.

### Clinical and Practical Implications

The M2M-C intervention has potential clinical and practical implications if proven successful. First, the M2M-C program would demonstrate the feasibility of delivering a structured, individualized tele-exercise platform. This approach effectively addresses common barriers faced by wheelchair users, such as transportation challenges, limited access to fitness facilities, and a lack of tailored exercise programs. The M2M-C program offers a convenient and accessible solution for engaging in physical activity from the comfort of one’s own home. Its adaptability makes it suitable for various settings, including rural or underserved areas where traditional exercise programs may not be available. The use of telecoaching also allows for regular interaction and personalized support, which are essential for maintaining motivation and long-term adherence. Moreover, if the M2M-C intervention proves successful, there is the potential for it to be expanded to serve a broader range of clinical populations. The program’s emphasis on individualization—adjusting exercises to align with each participant’s physical capabilities and fitness level—makes it adaptable. With appropriate modifications, the M2M-C program could be extended to other groups, including individuals with different types of mobility impairments and older adults with physical disabilities. This flexibility highlights the M2M-C intervention as a versatile tool for promoting physical activity on a larger scale, potentially reaching diverse populations in need of accessible exercise solutions.

## Appendix

Multimedia Appendix 1 Peer review report from the National Institute of Child Health and Human Development Special Emphasis Panel Home and Community-Based Physical Activity Interventions to Improve the Health of Wheelchair Users (National Institutes of Health, USA).

## Abbreviations

BP: blood pressure
CHIME: Cardiometabolic Health Intervention Using Music and Exercise
CMH: cardiometabolic health
FEV1: forced expiratory volume over 1 second
FVC: forced vital capacity
HbA1c: hemoglobin A1c
HR: heart rate
hsCRP: high sensitivity C-reactive protein
M2M: movement to music
M2M-C: movement-to-music cardiovascular
PI: principal investigator
PROMIS: Patient-Reported Outcomes Measurement Information Systems
RCT: randomized controlled trial
REDCap: Research Electronic Data Capture
RPE: rate of perceived exertion
SCI: spinal cord injury
SET: standard exercise training
SPIRIT: Standard Protocol Items: Recommendations for Interventional Trials
VO2peak: peak oxygen consumption

## Data Availability

The data generated as part of this study will be made available through a data repository. Additionally, deidentified datasets will be made available as supplementary material with each relevant publication.
