# Supplementary material for: Cardiometabolic Health Intervention Using Music and Exercise (CHIME) Delivered via Telehealth to Wheelchair Users: Protocol for a Randomized Controlled Trial
Source: JMIR Res Protoc. 2025 Jan 15;14:e57423. doi: 10.2196/57423 (PMC11780300; doi:10.2196/57423)
Supplement: Multimedia Appendix 1 [file resprot_v14i1e57423_app1.pdf]

1R01HD111059-01 Wilroy, Jereme

EARLY-STAGE INVESTIGATOR
NEW INVESTIGATOR

RESUME AND SUMMARY OF DISCUSSION:
This study seeks to examine the efficacy of a synchronous, telehealth Movement-to-Music (M2M) program on cardiometabolic health in wheelchair users. It is implementing a previously developed protocol in a well-designed trial. The proposal is well thought out, and the study team is well-suited to complete this study. If effective, the team has an established means to translate the program to reach users, rather than this being simply an academic exercise. The synchronous modality may have some cost and logistical challenges to implementation, but an asynchronous version will also be tested. And both present options that counter the many challenges faced by arm ergometry. There are some methodology details that could be improved, in terms of the behavioral intervention and details surrounding defining the protocol intensity. The reviewers have placed this application in the outstanding range.

DESCRIPTION (provided by applicant): For the more than 5.5 million people in the U.S. who use wheelchairs as their primary mode of ambulation, there is a pervasive lack of research on reducing cardiometabolic risk through structured exercise. Confirmatory analyses have revealed that wheelchair users live predominantly sedentary lifestyles and have substantially higher cardiometabolic risk factors compared to the general population. Although few exercises training studies have investigated cardiometabolic risk in certain subgroups with a disability, small sample sizes and homogeneous groups limit the translatability of this knowledge into clinical practice risk reduction strategies for wheelchair users. To address these issues, we propose to use a robust remote training system with built-in videoconferencing and real-time monitoring of vital sign data (e.g., heart rate, respiratory rate). This procedure will allow us to examine the efficacy of an evidence-based Movement-to-Music (M2M) program adapted for telehealth delivery and cardio emphasis (M2M-C). We are proposing a two-arm randomized controlled trial including 132 wheelchair users with a poor cardiometabolic profile (e.g., elevated triglycerides) recruited through our established network of clinics serving this population. The long-term goal of this proposal is to develop an effective and enjoyable modality for promoting health-enhancing exercise for wheelchair users by confirming exercise dose requirements for this underrepresented group. The primary aim will examine the average treatment effects of a 24-week M2M-C program on core indicators of CMH in wheelchair users with ≥ 2 cardiometabolic risk factors. CMH outcomes will be measured via blood tests (i.e., high sensitivity C-reactive protein, hemoglobin, fasting insulin, triglycerides, and cholesterol) and DEXA scan (i.e., body composition) at baseline and after 12 and 24 weeks of synchronous M2M-C training. The secondary aim will explore the beneficial effects of M2M-C on cardiovascular capacity, physical activity, and quality of life. Cardiovascular capacity will be measured via peak oxygen consumption. Physical activity and quality of life will be measured by self-report instruments validated for this population. The tertiary aim 1 is to evaluate the sustained effects of M2M-C (24 to 36 weeks) on physical activity. After M2M participants complete the 24-week program, they will be instructed to perform asynchronous exercise training using guided M2M online videos for an additional 12 weeks. The tertiary aim 2 is to examine the heterogeneity of treatment effect (HTE), which aims to understand whom the intervention is most effective.

PUBLIC HEALTH RELEVANCE: There are limited exercise options available for wheelchair users to improve their health. The major goal of this study is to test the efficacy of a live, synchronous exercise program with real-time monitoring of vital sign data to improve cardiometabolic health outcomes in

wheelchair users. The movement-to-music-cardio (M2M-C) intervention has the potential to improve the way exercise interventions are delivered to wheelchair users, subsequently leading to an increase in sustainable and scalable solutions to improving health outcomes in this population.

CRITIQUE 1

Significance: 1
Investigator(s): 1
Innovation: 2
Approach: 3
Environment: 1

Application #: **1 R01 HD111059-01**

Overall Impact: This proposal addresses a critical knowledge gap relating to exercise in wheelchair users by looking at changes in cardiometabolic risk factors in subjects at risk related to exercise. This information will be highly valuable in future clinical trials and clinical exercise prescription. There is a highly qualified team with diverse experience led by a PI who is experienced in the area and a person with relevant lived experience. There is an additional team member who is a PWLE. The protocol is innovative, tightly designed, described in great detail and addresses important barriers to exercise. There are a few issues to consider in the approach; perhaps the only one that is more than negligible is addressing potential pregnancy and DEXA.

1. Significance:

Strengths

- Focus on cardiometabolic health response to a homebased, equipment-light exercise program for wheelchair users, addressing a pressing issue in understanding proper exercise prescription for them.
- Multiple interventions to address compliance and maintenance that can be easily translated should they be successful.

Weaknesses

- None identified

2. Investigator(s):

Strengths

- Highly experienced team with diverse expertise
- PI has experience with trials of the intervention, and is a person with relevant lived experience (as is another team member)
- Identified clinician partnerships for recruiting

Weaknesses

- None identified

3. Innovation:

Strengths

- The selection of individuals with identified cardiac risk factors to better assess change with exercise
- The calculation of the exercise dose designed to lead to effects on risk factors, based on a meta-analysis
- The home monitoring during exercise
- The concept of achieving moderate levels of exercise with upper extremity-based movement and minimal equipment

Weaknesses

- Tele-exercise to music with social interaction is listed as an innovation; this is more of a cultural standard

4. Approach:

Strengths

- Strong recruiting strategies and populations, with experience, and in a setting that is a national leader for physical activity in disability
- Exercise in the home setting, low equipment needs (no arm ergometry!) will markedly increase compliance
- Well-described strategies for assuring safe participation
- Adequate length of follow up to measure real change in primary variables
- Analysis plan will look at dose response and aspects that will help personalize program

Weaknesses

- For tertiary aim 2, a deeper dive into variables such as severity of motor impairment, diagnosis, and other factors would be useful
- To make a better comparison, the control group should get some exercise advice, if not a prescription. Although admittedly not done as often as it should be, some discussion of exercise should be part of everyone's health care.
- Many of the participants will be ambulatory in part, and some will at least have lower extremity movement. It would seem to be problematic to focus only on upper extremity movement in this group over a 24-week period, without addressing the lower extremities.
- Hand weights and/or weighted sleeves are mentioned a few times in the proposal, but there is no mention of these being part of the equipment the subjects receive.
- The study population will include subjects who may be pregnant and does not discuss how this will be addressed in relation to DEXA scanning.

5. Environment:

Strengths

- Extremely strong clinical and research environment with much experience in this area and this type of research

Weaknesses

- None noted

Study Timeline:

Strengths

- Appropriate

Weaknesses

- None noted

Protections for Human Subjects:

Unacceptable Risks and/or Inadequate Protections

- The issue of possible pregnancy and DEXA must be addressed

Data and Safety Monitoring Plan (Applicable for Clinical Trials Only):

- Acceptable
    - No issues

Inclusion Plans:

- Sex/Gender: Distribution justified scientifically
- Race/Ethnicity: Distribution justified scientifically
- For NIH-Defined Phase III trials, Plans for valid design and analysis: Scientifically acceptable
- Inclusion/Exclusion Based on Age: Distribution justified scientifically
- Acceptable

Vertebrate Animals:

- Not Applicable (No Vertebrate Animals)

Biohazards:

- Not Applicable (No Biohazards)

Applications from Foreign Organizations:

- Not Applicable (No Foreign Organizations)

Select Agents:

- Not Applicable (No Select Agents)

Resource Sharing Plans:

- Acceptable

Authentication of Key Biological and/or Chemical Resources:

- Not Applicable (No Relevant Resources)

Budget and Period of Support:

- Recommend as Requested

CRITIQUE 2

Significance: 2
Investigator(s): 1
Innovation: 1
Approach: 2
Environment: 1

Application #: **1 R01 HD111059-01**

Overall Impact: This study seeks to examine the efficacy of a synchronous, telehealth Movement-to-Music (M2M) program on cardiometabolic health in wheelchair users. It is implementing a previously developed protocol in a well-designed trial. The proposal is well thought out, and the study team is well-suited to complete this study. If effective, the team has an established means to translate the program to reach users, rather than this being simply an academic exercise. The synchronous modality may have some cost and logistical challenges to implementation, but an asynchronous version will also be tested. And both present options that counter the many challenges faced by arm ergometry. There are some methodology details that could be improved, in terms of the behavioral intervention and details surrounding defining the protocol intensity.

1. Significance:

Strengths

- There is a lack of evidence supporting physical activity as a means to improve cardiovascular health in wheelchair users. This trial seeks to address this gap by identifying an effective dose of exercise and confirming the benefit.
- Promotes a more holistic exercise modality than arm ergometry, which solves some barriers to exercise presented by arm ergometry.
- Successful completion will produce an exercise intervention that may be readily accepted and implemented, can be simplified (has potential to be done using existing personal equipment), and may produce cardiovascular improvements.

Weaknesses

- Despite the importance of developing effective dosing, there is limited discussion about the effective dose. For the trial, calibration of dosing will occur in the lab prior to beginning the trial. How will that translate to a fully in-home intervention?
- The initial exercise modality uses 1-on-1 training. That is more difficult and expensive to obtain – what is the likelihood of broad translation?
- Little is known about this type of intervention and whether it will produce cardiovascular benefits across a large population. Preliminary data demonstrated effectiveness, but only on 3 men with SCI. A larger population may find it difficult to reach sufficient intensity to produce positive results.

2. Investigator(s):

Strengths

- Very strong research team.
- Lived experience team, plus two investigators who are wheelchair users themselves.

Weaknesses

- PI has less experience managing grants on this scale but has a strong support team and sufficient experience in Co-I roles.
- Many of the investigators have minimal involvement by time. The small amount of time may be too small for significant contributions.

3. Innovation:

Strengths

- Novel exercise program that does not suffer from the limitations of arm ergometry.
- The research team presents plans for expansion and translation of the protocol.

Weaknesses

- The study is an incremental change from previous work by the group. On the other hand, this provides an increased likelihood of success.

4. Approach:

Strengths

- The trial aims to recruit a large number of subjects, and previous studies from this team suggests they should have success with enrollment.
- The intervention is driven by behavioral change theory, potentially increasing the likelihood of adoption by wheelchair users.
- The inclusion criteria are strict in that they are limited to a higher risk population of wheelchair users. While this might make recruitment a challenge, they are the group most in need of the intervention.
- A variety of outcome measures associated with cardiovascular risk are proposed: C-reactive protein, HbA1C, fasting insulin, fasting triglycerides, fasting cholesterol, BP, and body composition.

Weaknesses

- The use of a behavioral support group may confound results. It is provided regularly to the intervention group and only twice to the control group. What if providing behavioral support would improve the participation in live and on-demand classes for the control group?
- More detail are needed about defining the exercise protocol intensity for each participant. How will VO2, heart rate and RPE data be integrated to make this decision? Also, it was mentioned that participants with blunted heart rate response will use a talk test, which was determined to be valid for vigorous exercise in the cited study. The goal in this intervention is moderate exercise – is there any evidence that the talk test will be valid? And how will this subgroup be identified?
- In a related concern, while heart rate is monitored continuously during activity to ensure that the correct intensity is maintained, how will the talk test be administered during each session?

- The involvement of persons with lived experiences was strong during the planning of the proposal. However, during this study, the involvement is limited to investigators, who have a stronger potential for bias as they are invested in the positive outcome of the intervention. Consider maintaining the involvement of a panel of persons with lived experiences throughout the study.
- How likely are individuals to know their personal health metrics for inclusion, and will exclusion by PAR-Q and fax to primary care physician work out with enough efficiency, given how difficult communications with health care providers can be?

5. Environment:

Strengths

- Lakeshore Research Facility is impressive

Weaknesses

- None noted

Study Timeline:

Strengths

- Looks reasonable

Weaknesses

- None noted

Protections for Human Subjects:

Acceptable Risks and/or Adequate Protections

- Although the risks are minor, they were not noted. No mention of the risks of physical activity to individuals in poor health who have not participated in exercise previously.

Data and Safety Monitoring Plan (Applicable for Clinical Trials Only):

- Acceptable
    - Adequate

Inclusion Plans:

- Sex/Gender: Distribution justified scientifically
- Race/Ethnicity: Distribution justified scientifically
- For NIH-Defined Phase III trials, Plans for valid design and analysis: Not applicable
- Inclusion/Exclusion Based on Age: Distribution justified scientifically
- Appropriate

Vertebrate Animals:

- Not Applicable (No Vertebrate Animals)

Biohazards:

- Not Applicable (No Biohazards)

Resubmission

- n/a

Renewal

- n/a

Revision

- n/a

Applications from Foreign Organizations:

- Not Applicable (No Foreign Organizations)

Select Agents:

- Not Applicable (No Select Agents)

Resource Sharing Plans:

- Acceptable

Authentication of Key Biological and/or Chemical Resources:

- Not Applicable (No Relevant Resources)

Budget and Period of Support:

- Recommend as Requested

CRITIQUE 3

Significance: 2
Investigator(s): 1
Innovation: 4
Approach: 3
Environment: 1

Application #: **R01 HD111059-01**

Overall Impact: This R01 from Dr. Wilroy at UAB tests the efficacy of telehealth delivered M2M-C intervention for improving cardiometabolic health among wheelchair users. Dr. Wilroy is a new and

early investigator with the training and experience necessary to lead this project with an exceptional team of Co-I’s in an exceptional environment. The proposed intervention has a strong history of development and testing which supports success of its implementation and potential for wide adoption. The overall protocol and plan is strong. While moderate to minor concerns exist in the power analysis, innovation, and feasibility, the enthusiasm remains high.

1. Significance:

Strengths

- Prior research and development on planned intervention (M2M-C) is strong and moves beyond only arm ergometry as chosen exercise modality.
- Home delivery of intervention overcomes barrier of time and transportation.
- Many factors support potential wide adoption including the telehealth, video-based nature of the intervention.

Weaknesses

- Preliminary data do not demonstrate potential for intervention to improve cardiometabolic outcomes.
- There is a call center for support however, unclear how scaling the 1:1 coaching aspects are for wide adoption.

2. Investigator(s):

Strengths

- Dr. Wilroy is a new and early-stage investigator with training and experience in exercise science, rehabilitation, and implementation science. He is well-prepared to lead the study with support of co-investigators.
- Dr. Rimmer has extensive experience and expertise in the proposed study protocol and leadership of similar studies. He can provide senior mentorship to Dr. Wilroy.
- Statistical analysis support provided by Dr. Oster.
- Strong support from experienced co-investigators across the necessary domains.

Weaknesses

- None noted

3. Innovation:

Strengths

- Remote training system with real-time monitoring of vital sign data

Weaknesses

- Physical activity measured with self-report only with no collection of objective daily physical activity patterns.
- Exercise through one modality only. No inclusion of daily activity as exercise.

4. Approach:

Strengths

- Broad suite of cardio and physical outcome measures including interviews.

- Strong statistical analysis plan including adequate description of potential moderators. Social determinants of health and self-efficacy may also be important moderators to collect.
- Providing access to exercise to controls that aligns with available exercise to population to test more pragmatic control.
- Broad inclusion with focus on high-risk individuals.
- Study design including maintenance period.
- Use of factors from behavior change wheel

Weaknesses

- Unclear justification in power analysis that expected changes/differences in groups is attainable or clinically meaningful.
- Justification is provided for the sufficient pool from which to recruit from across the 4 sites; however, justification from previous studies was not provided to demonstrate the enrollment rates from a similar population. Further, unclear from the total population who would qualify based on high-risk inclusion.
- Hypotheses for aims 1 and aims 2 are expected and simple without including known moderators of response including self-efficacy.
- Lack of discussion of how implementation outcomes will be assessed and used in analysis including adherence and compliance.

5. Environment:

Strengths

- Very strong environment at UAB including all the facilities, institutional support, resources, and equipment required to complete the proposed project successfully.

Weaknesses

- None noted

Study Timeline:

Strengths

- Data management is described including using existing resources.
- Detailed task/deliverables operational list demonstrates strong preparation.

Weaknesses

- Unclear if planned enrollment rate is feasible based on prior studies.

Protections for Human Subjects

Acceptable Risks and/or Adequate Protections

- There is prevention for overuse injuries built into the protocol; however, they are not mentioned as a potential risk from the intervention. There are no risks described except based on confidentiality with no reference to risk from exercise.

Data and Safety Monitoring Plan (Applicable for Clinical Trials Only):

- Acceptable

Inclusion Plans:

- Sex/Gender: Distribution justified scientifically
- Race/Ethnicity: Distribution justified scientifically
- For NIH-Defined Phase III trials, Plans for valid design and analysis: Scientifically acceptable
- Inclusion/Exclusion Based on Age: Distribution justified scientifically
- Strong justifications provided for sex/gender and race/ethnicity and across the lifespan with adequate ability to include women and minorities.

Vertebrate Animals:

- Not Applicable (No Vertebrate Animals)

Biohazards:

- Not Applicable (No Biohazards)

Applications from Foreign Organizations:

- Not Applicable (No Foreign Organizations)

Select Agents:

- Not Applicable (No Select Agents)

Resource Sharing Plans:

- Acceptable

Authentication of Key Biological and/or Chemical Resources:

- Not Applicable (No Relevant Resources)

Budget and Period of Support:

- Recommend as Requested

THE FOLLOWING SECTIONS WERE PREPARED BY THE SCIENTIFIC REVIEW OFFICER TO SUMMARIZE THE OUTCOME OF DISCUSSIONS OF THE REVIEW COMMITTEE, OR REVIEWERS' WRITTEN CRITIQUES, ON THE FOLLOWING ISSUES:

PROTECTION OF HUMAN SUBJECTS: ACCEPTABLE

INCLUSION OF WOMEN PLAN: ACCEPTABLE

INCLUSION OF MINORITIES PLAN: ACCEPTABLE

INCLUSION ACROSS THE LIFESPAN: ACCEPTABLE

Response to Reviewers' Comments
We appreciate the reviewers' thoughtful and detailed comments and have carefully considered each. As a result, we have provided responses to the noted weaknesses and believe the critiques have improved our proposal. Noted strengths in the application include the strong investigative team and environment, addressing a critical gap in cardiometabolic outcomes of exercise for wheelchair users, and a well-designed trial with strong potential for translation. The noted weaknesses related to two human subject concerns, adapting exercise training based on individual functioning, controlling for exercise dosing across sample, and statistical considerations. We have addressed each of these in the responses below.

Protection of Human Subjects
To ensure DEXA scans are not performed on pregnant individuals, we will ask women of child-bearing potential to complete a pregnancy test (using a urine sample) prior to each scan (baseline, 12-week, and 24-week post-intervention). If a pregnancy test is positive, DEXA scan will not be performed. Concerning the risk for participants who have not participated in physical activity previously, we include language in our consent form concerning the issue of muscle pain and soreness due to exercise. In addition, part of our exercise training protocol asks participants about current physiological states (e.g., pain) at the start of each session.

Approach
We will utilize V02 peak testing, RPE, and heart rate data from baseline testing to establish the exercise training intensity level for each participant. This involves using these data to calculate exercise training zone (i.e., light, moderate, vigorous) and inputting them into each participants' profile in the online platform. This allows for a convenient display of each participant's training zone during the exercise session. During each session we will collect objective data on heart rate and breathing rate. To address exercise dosing for individuals with blunted heart rate, we will include additional indicators for exercise intensity, including rate of perceived exertion (RPE) and the talk test. We will include RPE protocols previously developed in individuals with blunted heart rate. Also, we will include procedures such as familiarization and periodically asking for RPE during exercise session. The talk test will be used as a secondary measure, primarily to ensure that participants stay in the moderate intensity training zone and that RPE is related to aerobic strain as opposed to anaerobic.

Exercise movements for individuals with lower limb functioning are incorporated into our Movement-to-Music Cardio (M2M-C) routines. Our previous trials using M2M were designed for a range of disability groups. Each participant will receive exercise training routines tailored to their abilities and level of fitness, which involves movement selection (i.e., use of lower limbs if able). Although we understand the value of obtaining objective physical activity data, we justified not including it in this study due to lack of valid measures available for wheelchair users and to reduce participant burden. The current intervention involves 3 times per week of supervised exercise training for 24 weeks and physical activity is not a primary outcome.

We would like to clarify that the intended behavioral support in this trial is to emphasize the connection between trainers and participants and to address potential health/technology usability issues that inhibit joining the exercise training session. This will be a different approach from the exclusively behavioral counseling intervention that emphasizes structured education. Similarly, we described the two checkup points for the self-guided exercise control group at week 12 and 24 to address potential issues with accessing the virtual exercise membership and using the technology. There will be no structured behavioral coaching and counseling provided by study staff. For physical activity prescription, control participants will receive all of their recommendations from the virtual exercise membership, if they choose to use it.

Although several investigators have minimal effort, they meet regularly with the PI due to collaborations across multiple projects and within research centers. The effort involved in the proposed project is adequate for their advisory role. Similarly, due to strong relationships with rehab physicians, obtaining medical clearance for exercise has been an efficient process in our previous studies. For approval, we have clear protocols to obtain medical clearance from each participant's physician and it hasn't been an issue in our previous studies because we assign one of our staff to calling the physician's office manager or nurse as a reminder. We will maintain involvement of individuals with lived experience outside of the investigators. We currently have a group of individuals with lived experience who work or visit Lakeshore Foundation regularly as members. We added to the project milestones a quarterly meeting with this group of individuals to continue to inform the project.

Statistical Analysis Plan

Our expected changes/differences should be attainable for our sample size based on our prior experience with adults with spinal cord injury and on the results of our conservative power calculation. The standard deviation for power calculation was obtained from our previous pilot study. Our results would be considered as clinically meaningful since they are similar to the suggested criteria for the definition of clinically significant improvement. “Clinically significant improvement” was defined for each risk factor based on the minimum change deemed clinically important; for our purposes, “clinically significant improvement” equates to the following: approximately 10% decrease in total cholesterol, 30% reduction in triglycerides, and 5% reduction in body fat from the beginning of care to the most recent measure.

Adherence and compliance data are logged on the online training platform. Adherence and compliance rates will be assessed as proportions and their corresponding exact 95% confidence intervals will be obtained. These rates will be obtained overall and separately by age, sex, body composition, and ethnicity. These rates will be compared separately for age group, sex, body composition, and ethnicity using the Chi-square test or Fisher’s exact test if the assumptions for the Chi-square test are not met.

We will consider the suggested outcomes as potential moderators and test effect modification by including appropriate interaction terms in our repeated measure of mixed models, including: severity of motor impairment, using a clinician-rated assessment (e.g., Functional Independence Measure, diagnosis (e.g., spinal cord injury vs. multiple sclerosis vs. stroke), psychosocial metrics specific to *readiness to change* (e.g., self-efficacy, outcome expectations, and goal setting skills) aligned with well-tested social cognitive theory constructs, and other factors, such as wheelchair use status [full-time vs. part-time], medication [use vs. non-use], and dietary intake [daily caloric intake <2155 kcal vs. 2155 kcal] as listed in the *Statistical Analysis Appendix.*
